# Comparison of the analgesic efficacy of spray and tablet flurbiprofen for pain after soft tissue surgery

**DOI:** 10.1590/1807-3107bor-2024.vol38.0108

**Published:** 2024-11-08

**Authors:** Cennet Neslihan Eroglu, Mehmet Nuri Yuksek, Sadi Elasan, Yusuf Rodi Mizrak, Busra Karaca

**Affiliations:** 1 Akdeniz University, Faculty of Dentistry, Department of Oral & Maxillofacial Surgery, Antalya, Turkey.; 2 Yuzuncu Yil University, Faculty of Dentistry Department of Oral & Maxillofacial Surgery, Van, Turkey.; 3 Yuzuncu Yil University, Faculty of Medicine, Department of Biostatistics, Van, Turkey.

**Keywords:** Flurbiprofen, Pain, Surgery, Oral

## Abstract

The aim of this randomized clinical study was to assess the comparative efficacy of flurbiprofen in tablet and spray formulations for postoperative pain management in oral soft tissue wounds undergoing primary closure while investigating the feasibility of achieving optimal analgesia with reduced dosage and risk. Forty patients who underwent epulis fissuratum and frenulum excision for pre-prosthetic surgery were randomly assigned to receive either tablet or spray forms of flurbiprofen. The lesion dimensions were measured preoperatively, followed by excision and primary closure. The tablet group received oral tablets containing 100 mg of flurbiprofen twice daily, whereas the spray group received an oral spray containing 0.25% flurbiprofen, administered as two sprays thrice daily. Postoperative pain was assessed using the Numerical Rating Scale (NRS) until the 7^th^ day. Lesion size, drug consumption, and rescue analgesic use were compared between the groups. There were no statistically significant differences in the lesion size between the groups. However, the mean NRS score in the spray group was significantly lower in the spray group compared to than that in the tablet group at 6^th^ hour postoperatively (p = 0.037). Significant differences favoring the tablet group were observed in the first three doses of the drug (p = 0.001). No patients required rescue analgesics. The spray formulation of flurbiprofen demonstrated effective and safe pain relief in oral soft tissue wounds undergoing primary closure, with no reported adverse effects.

## Introduction

Postoperative pain is an acute pain pattern with the potential to become chronic in the postoperative period due to existing pathology, surgical procedures, or both. Postsurgical pain should be relieved as quickly and effectively as possible to support the healing process and rehabilitation and prevent complications.^1^

Nonsteroidal anti-inflammatory drugs (NSAIDs) are the preferred agents for managing pain and the inflammatory process in the postoperative period. However, NSAIDs need to be prescribed with caution due to their undesirable side effects on the gastrointestinal system and kidneys, bleeding time, and interactions with other drugs.^2,3^ Recent studies have suggested that NSAIDs may impact the gene expression of osteoblasts, potentially leading to undesirable effects on osteoblast and bone-forming capacity.^4,5^ Therefore, while alternative treatment methods are being investigated, topical application is recommended to reduce systemic exposure and ensure maximum drug concentration at the target tissue. Individuals with reduced CYP2C9 activity (CYP2C9 poor metabolizers) experience enhanced exposure to flurbiprofen due to decreased metabolic clearance of few NSAIDs, such as flurbiprofen, resulting in abnormally high drug plasma levels and an increased risk of side effects.^7^ In such cases, dose reduction is often necessary to prevent adverse effects. Globally, the frequency of alleles responsible for adverse drug reactions related to CYP2C9 is significantly high and cannot be overlooked.^8^

Flurbiprofen, a propionic acid derivative, is classified as a nonsteroidal anti-inflammatory drug (NSAID) that has peripheral effects similar to those of ibuprofen, naproxen, fenoprofen, and ketoprofen, all of which belong to the same pharmacological group.^9^ Like other NSAIDs, it inhibits the formation of products of the arachidonic acid cascade. Flurbiprofen is available in tablet, micropellet capsule, patch, topical spray, gel, mouthwash, and oral spray forms. Different forms of flurbiprofen achieve different drug concentrations at the target tissue.^10^ The oral spray is absorbed directly from the oral mucosa, achieving peak plasma concentrations within 1.5–2 hours. Its half-life is approximately 3–4 hours. In contrast, the tablet form of flurbiprofen contains 100 mg of the active ingredient. The oral spray, with a volume of 30 ml, contains 75 mg of the active ingredient. Each spray delivers 0.2 ml of solution, equivalent to 0.50 mg of flurbiprofen. For this reason, it is argued that with each dose of the oral spray form of flurbiprofen, the patient is exposed to a lower amount of the drug and, therefore, experiences fewer side effects.^9,11,12^ Compared with placebo, favorable results were reported for using flurbiprofen spray for postoperative pain in the secondary wound healing process.^13,14^ However, it has also been reported that flurbiprofen spray negatively affects the epithelialization of secondary wounds, and the drug's application on direct open wounds does not contribute to healing.^13^

In oral surgery, soft tissue surgery is usually performed on young or elderly individuals for orthodontic and prosthodontic indications. Drug selection for this group of patients is important in terms of side effects and unnecessary systemic drug burden. In this study, we examined whether the spray form of flurbiprofen yields effects similar to those of the tablet form in alleviating pain after oral surgery, aiming to address whether optimal analgesia can be attained with minimal dosage and risk.

## Methods

### Study pattern

The study population consisted of all patients referred for pre-prosthetic surgery of soft tissues at the Faculty of Dentistry, Department of Oral and Maxillofacial Surgery. The procedures were explained to the participants, and all of them signed consent forms before participating in the study. The study was approved by the Clinical Research Ethics Committee of the Faculty of Medicine at Yuzuncu Yil University (05/25102023) and submitted for clinical trial registration (NCT06238154). All procedures were performed in accordance with the 1964 Helsinki Declaration and its later amendments.

Given that those using removable prostheses had discontinued using the prosthesis one month prior (to minimize the lesion to be excised), healthy or ASA Class I patients between 40 and 65 years of age who had indications for preprosthetic surgery due to excision of the epulis fissuratum and frenulum, were included in the study.

The exclusion criteria were pregnancy, lactation, and taking contraceptive pills; being allergic to the drug or other NSAIDs to be used in the study; having used steroids or analgesic drugs for any reason in the last month; using psychiatric drugs; having incomplete data or refusing to sign the consent form; being extremely afraid or having a gag reflex; having any gastrointestinal problems; and smoking, ASA-2, and ASA-3 patients.

### Study groups

**Figure 1:**
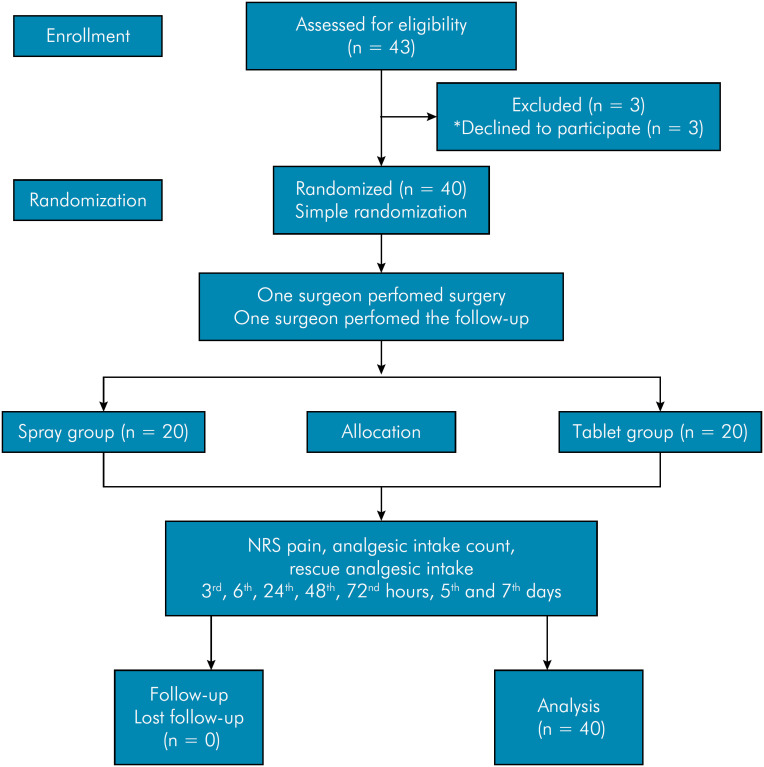
Consort diagram of the study.

The patients were divided into two groups, with 20 patients each in the flurbiprofen spray and flurbiprofen tablet group, by a simple randomization method (Figure 1). In the tablet group, flurbiprofen 100 mg tablets were prescribed to be taken twice daily with a 12-hour interval. In the spray group, flurbiprofen oral spray containing 0.25 mg was prescribed as two pumps for each use, three times a day, 8 hours apart. Patients were instructed to start using the medication when they felt pain.

### Surgical intervention

The operations were performed by a single oral and maxillofacial surgeon under local anesthesia using 80 mg of articaine hydrochloride with 0.02 mg of epinephrine. The incision borders were determined on healthy mucosa 2 mm away from the tissue to be excised, and elliptical incision lines were created with a No. 15 scalpel. The tissue to be excised was removed by dissection, starting from the distal part of the epulis fissuratum and the superior part of the frenulum. Hemostasis was achieved in the area. The wound was measured along the longest edge, and the measurement was recorded. Primary closure was performed with simple 4-0 silk sutures, and packing was applied. Tissues excised with the preliminary diagnosis of epulis fissuratum were sent for histopathological examination in formaldehyde solution, and the preliminary diagnosis was confirmed.

### Postoperative care

As analgesics, patients were given flurbiprofen and chlorhexidine gluconate + benzydamine HCl mouthwash (twice a day with a 12-hour interval for seven days) according to their groups. In addition, 500 mg of paracetamol was used as a rescue analgesic if needed. The patients were educated on the maximum number of sprays and the maximum number of tablets that could be used, as well as rescue medication used to relieve pain. Specifically, the tablet form should be taken on a full stomach and should not exceed four doses per day, and the spray form should not exceed two sprays thrice a day and should not be swallowed.

### Evaluated parameters

In the postoperative follow-up, pain was scored using the Numerical Rating Scale (NRS) at 3, 6, 24, 48, and 72 hours, and on the 5^th^ and 7^th^ day, the number of sprays, the number of tablets used, and the number of rescue medications used daily were recorded. Using the NRS, pain intensity was assessed on a 0-10 ranking scale, with 0 representing "no pain" and 10 representing "most severe pain".^15^ The investigators who collected and analyzed the parameters assessed were blinded.

### Statistical analysis

When the sample size of this prospective study was calculated, the power of the test for each variable was determined to be at least 80%, with a Type-1 error of 5%. Sample size calculation based on a previous study ^10^, determined that there should be a minimum of 15 patients in each group.

Shapiro-Wilk (n < 50) and Skewness-Kurtosis tests were used to check whether the continuous measurements in the study were distributed normally. Since the measurements showed a normal distribution, parametric tests were used. Descriptive statistics for continuous variables are expressed as the mean, standard deviation, number (n), and percentage (%). "Independent t-test" was used to compare continuous measurements according to group. The level of statistical significance was set at p < 0.05, and the IBM SPSS Statistics for Windows, Version 26.0 (IBM Corp., Armonk, USA), was used for analyses.

## Results

The tablet group comprised seven males and 13 females, with a mean age of 52.15 ± 7.4, and the spray group comprised seven males and 13 females, with a mean age of 48.95 ± 10.80 years. The epulis fissuratum and frenulum constituted 60% and 40% of the tissues. Statistical analysis of the mean lesion size across the groups is shown in Table 1. The mean operation time (from incision to final suture) was 10.36 ± 4.41 minutes for the spray group and 11.41 ± 3.29 minutes for the tablet group.

**Table 1:** Lesion sizes (length and width) in the study groups

Variables	Flurbiprofen spray (n = 20)		Flurbiprofen yablet (n = 20)		p-value*
	Mean	Std. Dev.	Mean	Std. Dev.	
Size L (mm)	20,2	11,265	26,35	13,758	0,13
Size W (mm)	8,1	3,959	10,55	3,913	0,056

Std. Dev.: Standart deviation;

* Independent t-test.

**Figure 2:**
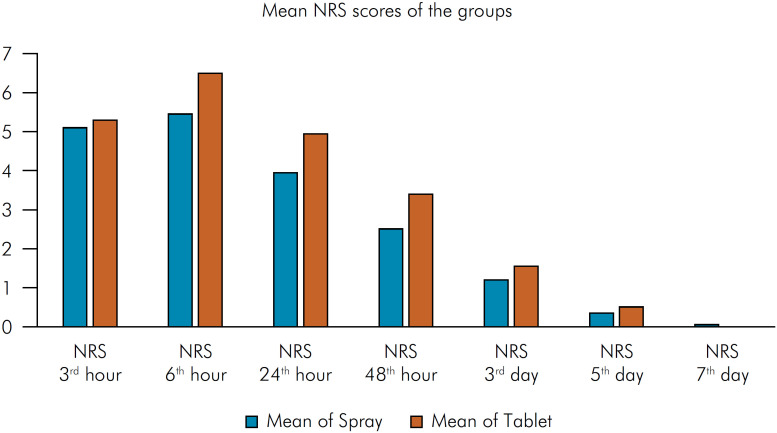
Mean NRS scores of the groups.

A comparison of the NRS scores between the groups revealed that the mean NRS score of the spray group at the 6^th^ hour was significantly lower than that of the tablet group (p = 0.037) (Figure 2). No statistically significant difference was observed at the other measured time points (p > 0.05) (Table 2).

**Table 2:** Comparison of mean NRS scores between groups

Variables	Flurbiprofen spray (n = 20)		Flurbiprofen tablet (n = 20)		p-value*
	Mean	Std. Dev.	Mean	Std. Dev.	
NRS 3^rd^ hour	5,1	1,68	5,3	1,3	0,677
NRS 6^th^ hour	5,45	1,7	6,5	1,36	0,037
NRS 24^th^ hour	3,95	1,76	4,95	1,47	0,059
NRS 48^th^ hour	2,5	1,67	3,4	1,63	0,093
NRS 3^rd^ day	1,2	1,43	1,55	1,39	0,439
NRS 5^th^ day	0,35	0,67	0,5	0,76	0,512
NRS 7^th^ day	0,05	0,22	0	0	0,324

Std. Dev.: Standart deviation;

* Independent t-test.

Regarding the patients’ drug intake, a significant difference was observed in favor of the tablet group (Table 3). None of the patients required rescue medication (Table 3). No side effects or recovery problems were encountered due to the medications use and the surgical procedures.

**Table 3:** The mean daily use of spray, tablet and rescue analgesics for each group.

Variable	Flurbiprofen spray (n = 20)		Flurbiprofen tablet (n = 20)		p-value*
	Mean	Std. Dev.	Mean	Std. Dev.	
Medication 1^st^ day	2,55	0,51	1,7	0,47	0,001
Medication 2^nd^ day	2,3	0,733	1,55	0,51	0,001
Medication 3^rd^ day	1,25	0,716	0,55	0,51	0,001
Medication 5^th^ day	0,15	0,366	0	0	0,075
Medication 7^th^ day	0	0	0	0	-
Rescue analgesic intake	0	0	0	0	-

Std. Dev.: Standart deviation;

* Independent t-test.

Regarding the amount of daily medication used according to the groups, medication intake continued on the 5th day in the spray group (Table 3). Regarding posology, the spray form met the patients’ pain complaints with an average of 3×1 sprays, whereas the tablet form met their painkiller need with a mean of 2 tablets per day (Table 3).

## Discussion

In the present study, we compared the effects of the tablet and spray forms of flurbiprofen, which significantly reduce the active ingredient content, on incisional wound pain in oral tissues. We aimed to investigate whether a lower amount of active ingredient can effectively control postoperative pain. The results favor the use of the spray form of the medication as it provides similar or better NRS data with a reduced amount of active ingredient. Despite the common preference among clinicians for tablet form of analgesics for postoperative pain control, our findings demonstrate that the active ingredient content in tablets is excessive for managing soft tissue surgery pain. We have established that successful postoperative analgesia can be achieved with the spray form of the active ingredient.

While it is expected that the use of rescue analgesics in patients using the tablet form would be less common than in patients using the spray form, our observation that none of the patients needed rescue analgesics is further proof of the adequate analgesic efficacy of the drug. The drug should be able to eliminate the need for rescue analgesics and demonstrate superiority over placebo.

When taken orally, the analgesic effect of flurbiprofen initially occurs within 30 minutes. Considering that the drug's activity occurs after gastrointestinal absorption, it is likely that the onset of analgesic activity will be shorter when the drug is applied directly to the wound surface. In a recent study, the effective time for the spray form of flurbiprofen for the mouth and throat regions was 20 minutes, faster than that of the lozenge (26 min) and granule (30 min) forms. With the aim of reducing pain as early as possible in mind, it was concluded that local absorption occurs faster than systemic absorption.^16^

In the study conducted by Dionne et al., which compared controlled-release flurbiprofen placed in the extraction socket with oral controlled-release flurbiprofen and placebo after impacted third molar extraction, found that peripheral use of the same dose of flurbiprofen was more effective than oral use.^17^ In another study by Battisti involving gingivectomy and root separation of molars, the reported pain intensity was similar in the 0.25% mouthwash form of the flurbiprofen group and tablet group.^18^ Our results are consistent with the literature. In peripheral application, absorption from the surgical wound and the desired analgesic effect in the adjacent area were comparable to those of the oral formulation, and the analgesic effect was observed for 3–6 hours. Delivering highly effective drug concentration to the target tissue prevents the distribution and elimination of the drug to other compartments. Additionally, although the blood level of the drug is lower in peripheral application, the analgesic effect be as good as high-dose oral flurbiprofen. Therefore, peripheral use of the same doses of flurbiprofen results in less exposure to the drug than oral use, hence reducing the risk of toxicity.^19^ In contrast, it has been reported that flurbiprofen has a higher concentration in soft tissue after topical application through the skin than after oral administration of equal doses.^10^ These findings suggest that although skin application of drugs is more effective than systemic applications^19^, topical applications carry a greater risk of local side effects at the same doses.

While considering the choices for route of administration for drug use, the blood circulation in surgical wounds can be utilized to minimize medication usage.^20^ In our study, the vascularization of the oral mucosa (compared with the skin, veins are closer to the surface, and there are many capillaries) and the primary closure of the wound created a smaller surface area, which is conducive to lower doses and fewer side effects. In another study comparing the spray form of flurbiprofen with placebo for post-tonsillectomy pain, it was shown that the topical use of flurbiprofen was superior to the placebo.^21^ In that study, the surgical area was not primarily closed. While postoperative pain is less intense in primary closed wounds, the contact surface of the applied topical agent is more limited. Similarly, Türk *et al*. reported that spray flurbiprofen + oral ibuprofen provided more effective analgesia than oral ibuprofen after tonsillectomy and that the spray form of flurbiprofen reduced the intensity of inflammation as of the 7^th^ day.^12^

In oral surgery, especially in procedures involving soft tissue surgery (such as incisional biopsies, pre-prosthetic surgery, soft tissue revisions, etc.), wounds are mostly the primary closed type, and pain management is provided with the use of systemic analgesics during the postoperative period. To the best of our knowledge, studies conducted using the spray form of flurbiprofen thus far consist of wound forms containing secondary healing sites.^12-14,22,23^ Different analgesic preferences, forms, and dose-related clinical outcomes should be discussed according to clinical cases, surgical approaches, and wound recovery models. The literature is still lacking in this context for many drugs. We believe our contribution to this area's literature will be significant.

The Numerical Rating Scale (NRS) is used to evaluate pain; it is a subjective scale that is affected by personal and environmental factors. The NRS is a valid and up-to-date method in which the patient can easily score the pain he or she feels and it is easy for patients to understand and use.^14,21 24,25^ Therefore, we chose to use this scale in our study, considering these advantages.

None of the flurbiprofen-related side effects reported in the literature were observed in any patient during this study. Although working with a small sample size is a limitation of the study, we obtained good results regarding low dosage and analgesic efficacy, especially considering the NRS data collected on the first postoperative day and at 6 hours. Another limitation of the study may be the lack of anti-inflammatory evaluation. However, the mouthwash prescribed to patients postoperatively may have influenced this evaluation. Given the variations in drug administration routes and concentrations, absorption rates from the oral mucosa and gastrointestinal tract naturally vary. Additionally, the drug's rate of metabolism and half-life are influenced by absorption, leading to inevitable differences in the frequency of daily administration.^26^

The surgical technique for excising the epulis fissuratum and frenulum was standardized as much as possible. However, the epulis fissuratum involved a full-thickness dissection, whereas the frenulum was treated with a half-thickness dissection. Since primary closure was achieved for all lesions, it is not expected to affect the postoperative pain significantly. However, differences in pain perception during cheek and lip movements may exist between these two regions, which may have influenced the results.

In summary, to minimize the potential for systemic side effects, we believe that the spray form of flurbiprofen is a preferable analgesic for incisional wounds in oral soft tissue, as it has analgesic efficacy similar to that of the tablet form. We argue that systemic analgesic administration after oral soft tissue surgery is unnecessary.

## Conclusion

The use of analgesic tablets results in an unnecessary drug burden in primary closed wounds after soft tissue surgery. Spray-form analgesics present a safe and effective option for pain management at much lower doses.
